# Clinical investigation in non-liver cirrhosis portosystemic shunt encephalopathy—four case series—

**DOI:** 10.20407/fmj.2020-008

**Published:** 2020-12-16

**Authors:** Fumika Azuma, Kazuya Nokura, Tetsuharu Kako, Yoshihiko Horimoto, Eiichi Katada, Naohide Kondo, Yasuhiro Ito

**Affiliations:** 1 Department of Neurology, Fujita Health University Bantane Hospital, Nagoya, Aichi, Japan; 2 Department of Neurology, Nagoya City Rehabilitation and Sports Center, Nagoya, Aichi, Japan; 3 Department of Neurology, Nagoya City West Medical Center, Nagoya, Aichi, Japan; 4 Department of Neurology, Toyota Memorial Hospital, Toyota, Aichi, Japan

**Keywords:** Portosystemic shunt, Encephalopathy, Hyperammonemia, Interventional radiology

## Abstract

We reported here four cases presenting with disturbance of consciousness over long periods of time and hyperammonemia. Two patients were on maintenance hemodialysis. Contrast-enhanced computed tomography (CT) of abdomen and balloon-occluded retrograde contrast venography revealed existence of a non-cirrhotic portosystemic shunt. Conservative treatment such as intravenous branched-chain amino acid administration and oral lactulose administration had only a modest effect in all patients. Improvements in symptoms were observed following the occlusion of the shunt path in three patients. Measurements of ammonia values would be the most important test for screening, but changes in Fischer’s ratio or indocyanine green (ICG) test values were also correlated with clinical symptoms. Neurologists should keep in mind the possibility of non-cirrhotic portosystemic shunts when they encounter patients with disturbance of consciousness. They should also remember that occlusion of the shunt pathway is an effective treatment.

## Introduction

Non-cirrhotic portosystemic shunts are a very rare condition. They are mostly limited to case reports in the field of neurology. Symptoms are similar to those of hyperammonemia-induced hepatic encephalopathy. Liver function tests almost always show no abnormalities, and the presence of a shunt is easily missed.^1^ Some reports have indicated that early diagnosis is difficult,^2^ and others have reported that it could sometimes be misdiagnosed as dementia.^3^ Patients may consult neurologists due to various neurological symptoms, including disturbance of consciousness. Hence those neurologists should be aware of the state of this disease. We would like to review the treatment course and neurological findings (before and after treatment) in our four patients to provide points of caution. Additionally, we would like to discuss the literature.

## Patients and Methods

The subjects were four patients with non-cirrhotic portosystemic shunts who were treated in our hospital and associated hospitals between 1988 and 2004. We retrospectively reviewed the patient’s neurological symptoms, laboratory tests, and clinical course. Of these four patients, one was reported in a case report in another journal,^4^ which we have reproduced here with permission.

## Results

### General background

Age, sex, past medical history, time from initial onset to treatment, and triggers that exacerbated disturbance of consciousness are summarized in Table 1. Ages ranged from 65 to 82 years, with an average of 73.5 years. Liver cirrhosis was not identified in general blood chemistry, abdominal ultrasonography, or upper abdominal computed tomography (CT) for all four patients. No liver biopsy was performed. The presence of a portosystemic shunt was evidenced by contrast-enhanced CT of abdomen and balloon-occluded retrograde contrast venography. Two of the four patients were on maintenance hemodialysis. Case 2 had a history of partial resection of the ascending transverse colon due to colon cancer, but the other patients had no history of abdominal surgery, including biopsy.

### Clinical course until hospital visit

The period in which disturbance of consciousness appeared and resulted in visits to our hospital and related hospitals ranged from three months to several years. Two patients underwent emergency hospital admission due to disturbance of consciousness. The course leading to hospitalization for each patient is shown below.

Case 1: A 65-year-old female had difficulty speaking and shaking hands when fatigued since 19XX, and dementia was suspected by the family. In 19XX+6 months, the patient could not converse after having gone out to help with housework and exhibited abnormal behavior such as suddenly singing songs and eating dinner by grasping at food. In 19XX+7 months, the patient had incontinence, wandered, and was subsequently admitted to the psychiatry department for approximately one month following a dementia diagnosis. Symptoms fluctuated and the patient cycled in and out of the hospital. However, the patient then suddenly experienced disturbances of consciousness and underwent emergency hospitalization in 19XX+15 months.

Case 2: An 82-year-old female with a history of colon cancer surgery was admitted to a local hospital with recurrent remission of psychiatric symptoms and disturbance of consciousness since around 200X. The patient was admitted to the hospital because of hyperammonemia during hospitalization. The patient was referred and hospitalized in 200X+3 months.

Case 3: A 73-year-old male started hemodialysis due to diabetic nephropathy in 200X–2 years. The patient was admitted to the nephrology department for disturbance of consciousness since 200X and was diagnosed with hyperammonemia of unknown origin. The patient exhibited dizziness, nausea, restlessness, and somnolence, even after being discharged from the hospital, and in 200X+3 months, the patient was referred and admitted to our hospital.

Case 4: A 74-year-old female started hemodialysis due to diabetic nephropathy in 200X–5 years. The patient subsequently cycled in and out of the hospital due to transient disturbances of consciousness and shunt problems. The patient was suspected to have disturbances of consciousness due to hyperammonemia caused by a splenorenal shunt, and follow-ups were being carried out with symptomatic treatment. Decreased levels of consciousness were observed following the end of hemodialysis in 200X, and temporary improvements were observed. However, conditions worsened the evening of the next day, oral intake became impossible and conjugate deviation of eyes to the left appeared after two days. Therefore, the patient was transferred from the hemodialysis hospital during hospitalization.

### Changes in neurological symptoms

The portosystemic shunt areas and changes in neurological symptoms before and after treatment in the four patients are summarized in Table 2. The degree of coma was according to the classification of impairment of consciousness in hepatic encephalopathy, as described in the code for handling portal hypertension.^5^ So-called flapping tremors were only exhibited in case 1. Electroencephalograms could be performed before and after the treatment in only two patients: in case 1, the multiple triphasic waves present before treatment disappeared after conservative treatment, and in case 4, θ waves which appeared on the whole cranium before treatment clearly decreased after treatment. Case 4 scored 10 points on the Hasegawa dementia rating scale-revised (cut-off value: 20/21) before treatment; the score improved to 27 points after treatment.

### Changes in blood chemistry results

**Figure 1:**
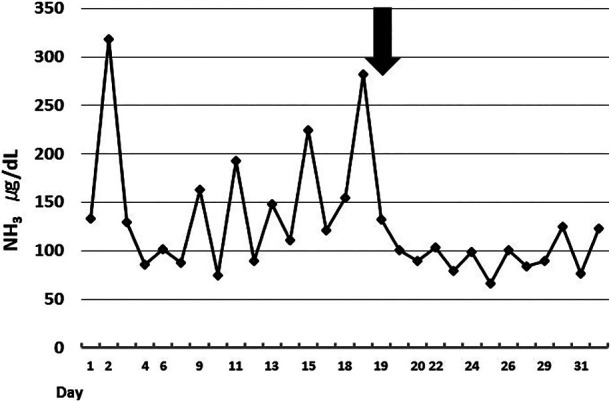
Changes in ammonia levels are shown for case 4. Large circadian variation is shown in the same disease one day before and one day after hemodialysis, and the ammonia level decreases after hemodialysis. Arrows in the figures indicate embolization. No apparent increases were observed after embolization.

As for test results, the ammonia levels at the time of admission were 247 μg/dL (normally 30–80 μg/dL) in case 1, 169 μg/dL in case 2, 435 μg/dL in case 3, and 318 μg/dL in case 4. All patients, excluding case 1, showed improvements to about 100–130 μg/dL at the time of hospital discharge. As for the Fischer’s ratio and indocyanine green (ICG) test results among patients for whom these tests could be conducted, Fischer’s ratio improved from 1.21 to 2.13 in case 4. This patient also showed an improvement from 41.8% to 26.8% (normally <10%) for the ICG test (corrected R15). Changes in ammonia level for case 4 are shown in Figure 1. Two tests were performed on the same day, before and after hemodialysis, and the ammonia level was normalized after hemodialysis. However, the ammonia level not only decreased, but also did not fluctuate after embolization (Figure 1).

### Course of treatment

**Figure 2:**
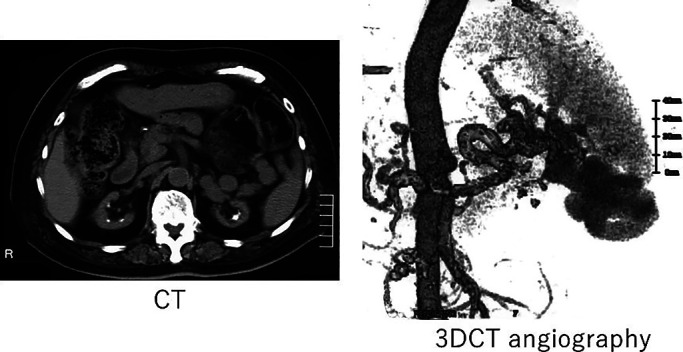
Plain abdominal CT and 3D CT angiography for case 4. Abnormal vessels were found from the splenic vein to the left renal vein in case 4.

**Figure 3:**
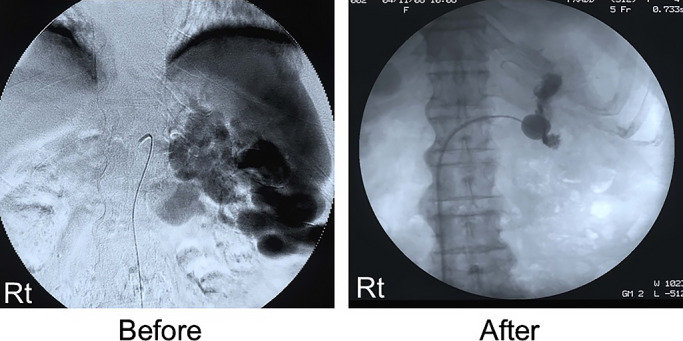
Angiography before and after balloon-occluded retrograde transvenous obliteration (B-RTO) in case 4. The abnormal vessel recognized before the treatment was not depicted by the embolization. Reprinted with permission of Ref. 4.

Case 1 underwent only conservative treatments such as intravenous branched-chain amino acid administration and oral lactulose administration, and no embolization was performed. Balloon-occluded retrograde transvenous obliteration (B-RTO) was performed on case 2 through 4 since conservative treatment alone was not sufficient; thereafter, symptoms improved. Conservative treatment was somewhat successful in all patients, but a recurrence of symptoms was observed, and clear improvements were observed after embolization (Figure 2 and Figure 3).

After several years of follow-up, the status of case 1 is unknown, but case 3 and 4 passed away due to infections and post-bone fracture complications, while case 2 is still under follow-up.

## Discussion

Classifying hepatic encephalopathy into types A (Acute type), B (Bypass type), and C (Cirrhosis type) according to the clinical course and mode of encephalopathy onset has been a recent trend in Western countries.^6^ Type A refers to acute hepatic failure (fulminant hepatitis). Type B refers to encephalopathy observed in portosystemic shunts and without liver disease (e.g., cirrhosis). Type C refers to encephalopathy observed in hepatic cirrhosis. The four patients investigated in this were classified as Type B. The causes for the development of portosystemic shunts seen in Type B patients were as follows: ① residual anastomoses of the embryonic portal vein and inferior vena cava,^7,8^ and ② complications after abdominal operations and/or trauma.^9^ Many reports of encephalopathy onset are at or after middle age,^1^ with a relatively high average age of onset in the present experienced cases, at 73.5 years. Even if caused by congenital vascular abnormalities, onset in these scenarios is thought to be due to the gradual development of the existing shunt due to the fragility of the blood vessel and hemodynamic changes with aging.

Results of a relatively large epidemiological study of portosystemic shunts in Japan reported that among 47 reported patients, 23 (48.9%) had extrahepatic shunts without portal hypertension, such as from the left gastric vein, splenic vein, and superior mesenteric vein to the left renal vein and inferior vena cava.^1^ Some of these patients were misdiagnosed with dementia or psychiatric disorders, and another report with 23 patients indicated that differentiation with dementia was difficult.^10^ Yet another report misdiagnosed patients with senile dementia during a three-year clinical course, with repeated onsets of disturbance of consciousness.^3^ As previously mentioned, patients in our report also had various clinical courses before hospitalization with no consistent findings regarding the degree of disturbance of consciousness or the duration of symptoms, which made it difficult to differentiate from dementia or symptomatic epilepsy. Fluctuating symptoms and findings are thought to be a point of differentiation with portosystemic shunts. Even patients with delayed diagnoses and a high degree of dementia are thought to have a possibility of improvement, and aggressive treatment is ideal.

Potential causative agents of hepatic encephalopathy include substances with low molecular weight, such as ammonia, amino acids, aminic acids, short-chain fatty acids, mercaptans, and gamma amino butyric acid (GABA) and substances with moderate to large molecular weights ranging from 5,000 to 50,000. However, except for ammonia, there are many unknowns regarding these substances. Prolonged hyperammonemia also leads to abnormalities in neurotransmitters, receptors, and the blood-brain barrier, which results in a disease state of increased sensitivity to ammonia and other toxic substances and increased susceptibility to encephalopathy.^11,12^ Clinical manifestations and blood ammonia levels are generally not correlated,^13^ and ammonia levels were poorly correlated with the detailed changes in the disease state, even in this case series. In particular, hemodialysis patients showed onset or exacerbation of disturbances of consciousness despite lower ammonia levels after hemodialysis. Therefore, it was thought that causative agents other than ammonia or complex changes in fluid volume or electrolytes due to hemodialysis were contributing, and no direct relationships between blood ammonia concentrations and psychological/neurological symptoms were observed.

Some case reports have indicated patients who exhibited disturbances of consciousness during chronic maintenance hemodialysis and where a shunt blood vessel was indicated.^14-18^ We also experienced two patients who exhibited disturbances of consciousness during maintenance hemodialysis and where diagnoses were made based on hyperammonemia and diagnostic imaging. There has been a hypothesis that patients with renal insufficiency have fluid overload and increased shunt flow with clinical progress, which in turn leads to further shunt development.^16^ Two out of our four patients were also on maintenance hemodialysis. The splenic and renal veins ran parallel, possibly through small anastomoses, and it was thought that renal blood flow decreased with progressive renal failure, which led to the development of a shunt due to differences in the decreased renal venous pressure and splenic venous pressure. Expanding differences in pressure due to hemodialysis may be an inducer of symptoms like disturbances of consciousness. It has also been previously indicated that the central venous pressure decreased due to rapidly decreased circulating blood volume due to hemodialysis, making blood flow easier from the portal vein to the central vein.^4^

Meanwhile, investigations based on the false neurotransmitter hypothesis, which has been proposed by Fischer et al. as a factor for decreased branched chain amino acids (BCAA) and increased aromatic amino acids (AAA), indicated that measurements,^19^ not only of ammonia, but also Fischer’s ratio, was effective when disturbances of consciousness due to a portosystemic shunt were present. ICG tests reflect hepatic function well. The rate of disappearance of ICG in blood is delayed when effective hepatic blood flow decreases or hepatocyte uptake increases. Our study only measured in case 4, but it is thought that measurements before and after embolization enable the tracking of changes in effective hepatic blood volume, and ICG tests are thought to be effective in determining effects before and after treatment. However, there have also been reports that patients who have been left untreated for long periods of time had not normalized due to decreased hepatic function caused by decreased hepatic blood flow,^20^ and determining this with Fischer’s ratio or ICG tests alone is not possible.

The interventional radiology (IVR) technique, which uses coils and balloons to embolize the shunt, has been increasingly used to occlude the shunt pathway beyond surgical operation. In particular, B-RTO is less invasive than other treatments and is becoming a first-line definitive treatment.^21^ A problem with long-term management of patients undergoing B-RTO includes the recanalization of thrombosed shunts, the creation of new shunts, and the need for continued careful follow-up after treatment.

Although non-cirrhotic portosystemic shunts are extremely rare, they should be considered a differential diagnosis in patients with varying degrees of consciousness and cognitive impairment. In these cases, the patients improved with the occlusion of the shunt. Therefore, it is crucial during screening to demonstrate hyperammonemia, and care must be taken because a small fraction of patients with disturbances of consciousness or floating cognitive dysfunction are referred from different departments to consult a neurologist.

## Supplementary Material

PDF-Japanese

## Figures and Tables

**Table1:** Patient characteristics

Case	Age	Sex	Past history and complications	Period from onset to treatment	Induce factor
1	65	F	None	A few	Over working
2	82	F	Colon cancer	Five months	None
3	73	M	Hemodialysis (diabetic nephropathy), Atrial fibrillation, cerebral infarction	Five months	Hemodialysis
4	74	F	Hemodialysis (diabetic nephropathy), cerebral infarction	A few	Hemodialysis constipation

F: female, M: male

**Table2:** Main shunt vessel and changes in consciousness before/after treatment

Case	Main shunt vessel	State of Consciousness (Before)	Treatment	State of Consciousness (After)
1	SMV-IVC	Coma II～III	Conservative	Coma I～II
2	SV-IVC	Coma II～III	B-RTO	Clear
3	SV-LRV	Coma II～III	B-RTO	Clear
4	SV-LRV	Coma II	B-RTO	Clear

SMV: superior mesenteric vein, IVC: inferior vena cava, SV: splenic vein, LRV: left renal vein, B-RTO: balloon-occluded retrograde transvenous obliteration
