# Supplementary material for: Clinical investigation in non-liver cirrhosis portosystemic shunt encephalopathy—four case series—
Source: Fujita Med J. 2020 Dec 16;7(4):139–42. doi: 10.20407/fmj.2020-008 (PMC8761820; doi:10.20407/fmj.2020-008)
Supplement: Supplementary file 1 — PDF-Japanese [file fmj-7-139-s001.pdf]

Case report

非肝硬変性門脈大循環短絡症により脳症をきたした症例の臨床検討-4 症例-

東　文香[1], 野倉一也[1], 加子哲治[1], 堀本佳彦[2], 片田栄一[3], 近藤直英[4], 伊藤　泰広[4]

1 藤田医科大学ばんたね病院　脳神経内科
2 名古屋市総合リハビリテーションセンター　脳神経内科
3 名古屋市立西部医療センター　脳神経内科
4 トヨタ記念病院　脳神経内科

要旨

非肝硬変性門脈大循環短絡症による高アンモニア血症を呈した 4 症例について臨床的に検討した．全例高アンモニア血症を認め，長期間の浮動性の意識障害を主訴とした. 2 例は維持透析患者であった．画像診断は腹部造影 CT とバルーン閉塞下経静脈造影によった．全例で内科的治療には効果は乏しく，3 例で短絡路の閉鎖によって症状の改善が認められた．アンモニア値測定がスクリーニングには最も重要であるが，Fischer 比や ICG 値の変化が臨床症状に相関した．浮動性の意識障害において神経内科医師は肝硬変症がない門脈大循環短絡症の可能性を念頭に診療し，短絡路の閉鎖が有効な治療法である事を銘記しておかなければならない.

Key words
Portosystemic shunt, Encephalopathy, Hyperammonemia, Interventional radiology

はじめに

非肝硬変性門脈大循環短絡症は，非常にまれな疾患であり，神経内科領域ではほとんど症例報告に限られる．症状は高アンモニア血症による肝性脳症と類似するが，検査上肝機能検査はほぼ正常を示し，短絡路の存在は見逃されやすく[1],早期診断は困難であるとの報告もあり[2], 認知症と誤診されていた報告もある[3]. 意識障害を始めとする種々の神経症状を示し神経内科にコンサルトされる可能性があり，神経内科医師が見逃してはいけない病態として周知しておくべきであり，われわれが経験した4症例について，治療経過，治療前後の神経所見を検討，若干の文献的考察を加え注意を喚起したい．

Ⅰ　対象および方法

対象は1988年から2004年の間に当病院および関連病院で入院治療した非肝硬変性門脈大循環短絡症を有した4症例であり，検査値，神経症状，臨床経過について後方視的に検討した．内1例はすでに他誌に症例報告として報告した[4]が内容の一部を許可を得て再掲した．

Ⅱ　結果

1)　一般背景

年齢，性，既往歴，初発から治療までの期間，意識障害増悪の誘因を表1にまとめた．年齢は65歳から82歳であり，平均73.5歳であった．全例で一般血液生化学検査，腹部超音波検査もしくは，上腹部CT検査で肝硬変の所見はなかった．肝生検は施行していない．門脈大循環短絡の存在は腹部造影CTと放射線科によるバルーン閉塞下逆行性経静脈造影によって証明された. 4例のうち2例が維持透析を受けていた．症例2は結腸癌のため上行横行結腸部分切除術の既往があったが，その他の症例には生検を含む腹部手術の既往はなかった．

2)　受診に至るまでの経過

意識障害が出現し，当院および関連病院に受診に至った期間は，3ヶ月から数年であり，意識障害で緊急入院をした症例が2例あった．以下に各症例の入院に至る経過を示す．

症例1: 65歳女性であり,19XX年から疲労時に話しづらさと，手のふるえがあり，家族は認知症かと考えていた．19XX年＋6ヶ月，家事の手伝いに遠方に出かけた折りに会話が通じなくなり，急に歌を歌いだす，手づかみで夕食を食べるなどの異常行動が出現した．19XX 年＋7ヶ月頃から失禁，徘徊があり，認知症の診断で約1ヶ月間精神科へ入院した．症状の変動がありその後も入退院を繰り返していたが，突然意識障害をきたし19XX年＋15ヶ月に緊急入院した．

症例2: 結腸癌の手術既往がある82歳女性であり, 200X年頃より再発寛解を反復する精神症状，意識障害にて近医入院し，入院中に高アンモニア血症が認められたため，200X年＋3ヶ月に紹介され入院した．

症例3 : 73歳男性であり, 200X－2年糖尿病性腎症により透析導入．200X年から意識障害のため腎臓内科に入院し，原因不明の高アンモニア血症と診断された．退院後もめまい，嘔気，不穏，傾眠を認め200X年＋3ヶ月紹介され入院した．

症例4 : 74歳女性であり, 200X－5年糖尿病性腎症により透析導入．その後，一過性の意識障害やシャントトラブルで入退院をくり返し，脾腎短絡が原因の高アンモニア血症による意識障害を疑われていたが，対症療法で経過観察中であった．200X 年透析終了後に意識レベルの低下を認め，一時改善したが，翌日の夕方から再び状態が悪化し，経口摂取が不能になり，2日後には左共同偏視が出現したため，入院中の透析医院から転院した．

3)　神経症候の変化

4 症例の門脈大循環短絡路領域と治療前後の神経症候の変化を表 2 にまとめた．肝性脳症の昏睡度については，門脈圧亢進症取扱い規約による肝性脳症の意識障害の分類に準じた[5]. いわゆる羽ばたき振戦を症例 1 のみ認めた．治療前後に脳波を施行できたのは 2 例で，症例 1 については，治療前に多発していた三相波が保存治療後に消失し，症例 4 については，治療前は全般性に出現していた θ 波が，治療後には明らかに減少を認めた．また，症例 4 については，スクリーニングのため行った長谷川式簡易知能評価スケール（カットオフ値: 20/21）が，治療前は 10 点だったが，治療後は 27 点に改善した．

4)　血液生化学検査結果の変化

検査結果においては，入院時のアンモニア値が症例 1 では 247μg/dl（正常 30～80μg/dL)，症例 2 では 169μg/dL，症例 3 では 435μg/dL，症例 4 では 318μg/dL であり，退院時は症例 1 を除いて，約 100～130μg/dL 程度に改善した．その他の施行できた症例での Fischer 比や ICG 値の検査結果においては，症例 4 では Fischer 比が 1.21 から 2.13 に改善を認めた．また症例 4 では ICG 試験 （補正 R15）が 41.8%から 26.8%（正常 10%＞）に改善した．症例 4 のアンモニア値の変化を図 1 に示す．同日に 2 回の検査されたのは透析前後であり透析後にはアンモニア値は低下をしているが，塞栓術後はアンモニア値の低下と相まって変動がなくなった（図 1)．

5)　治療経過

症例 1 は分子鎖アミノ酸製剤，ラクチュロースを用いた保存療法のみで塞栓術は施行していない．症例 2 から 4 は保存療法だけでは不十分でバルーン閉塞下逆行性経静脈的塞栓術（balloon-occluded retrograde transvenous obliteration;B-RTO）を施行し，症状の改善を認めた．全例で保存療法は多少奏功したが，症状の再発を認め，塞栓術によって明らかな改善をみた（図 2，図 3)．

数年の経過観察後，症例 1 は不明であるが，3，4 については感染症や骨折後合併症にて死亡，症例 2 については引き続き経過観察中である．

Ⅲ　考察

最近欧米では、肝性脳症を臨床経過や脳症の発症様式により A 型（Acute type)，B 型（Bypass type)，C 型（Cirrhosis type）に分類する傾向にある[6]．A 型は急性肝不全（劇症肝炎）でみられる脳症，B 型は門脈大循環短絡でみられる脳症で肝硬変などの肝疾患を伴わないもの，C 型は肝硬変でみられる脳症を指す．今回検討した 4 症例は B 型に該当する．B 型の症例での Portosystemic shunt が発達する原因としては，①胎生期の門脈と下大静脈系吻合の遺残[7, 8]，②腹部手術や外傷後の合併症[9]が考えられている．脳症の発症の報告の多くは中年期以降であり[1]，今回の経験例も発症平均年齢は 73.5 歳と比較的高齢者であり,先天性の血管異常が原因であっても，もともと存在した短絡路が加齢に伴う血管の脆弱性や血行動態の変化などにより，徐々に発達したためと考えられる．

国内においての門脈大循環短絡路症の比較的大規模な疫学調査の結果，47 症例の報告のうち，門脈圧亢進を伴わない左胃静脈，脾静脈，上腸間膜静脈から左腎静脈や下大静脈へなどの肝外短絡路を有する症例は 23 症例（48.9%）であった[1]．これらの症例の中には認知症や精神科疾患と誤診されていた例もあり，別の 23 症例をまとめた報告でも，認知症との鑑別が困難であると記してある[10]．他にも 3 年の経過で老人性認知症と誤診され，意識障害の発作を繰り返していた症例の報告もある[3]．われわれの症例も

前述したとおり，入院前の経過は様々で，意識障害の程度や症状の変動もその期間も一定した所見はなく，認知症や症候性てんかんとの鑑別が困難であった．鑑別点として，門脈大循環短絡症では症状や所見が変動することが特徴であると思われる．さらに診断が遅れ，高度の認知症を有すると思われる症例でも改善する可能性があり，積極的な治療が望まれる．

肝性脳症を引き起こす起因物質としてはアンモニア，アミノ酸，アミン酸，短鎖脂肪酸，メルカプタン，GABA などの低分子量物質から，5,000－50,000 程度の中－大分子量物質も想定されているが，アンモニア以外の物質については不明な点も多い．また，長期にわたる高アンモニア血症により，神経伝達物質，受容体，血液脳関門の異常が生じ，アンモニアなどの中毒物質に対する感受性が亢進し，容易に脳症を引き起こしやすい病態が生じている [11, 12]．一般的に臨床症状と血中アンモニア濃度は必ずしも相関せず [13]，本症例シリーズでもアンモニア値は詳細な病状の変化との相関関係は比較的乏しかった．特に透析患者では，透析後にアンモニア値が低くなったにも関わらず，意識障害が発症したり増悪したりしており，アンモニア以外の起因物質の関与や透析による体液量や電解質の変化などが複雑に関与していると考えられ，血中アンモニア濃度と精神神経症候にはリアルタイムでの直接関係は指摘できなかった．

慢性維持透析中に意識障害を認め，シャント血管を指摘された症例は数例報告が散見され [14-18]，我々も維持透析中に意識障害を発症し, 高アンモニア血症, 画像診断から診断に至った症例を 2 例経験した．腎不全患者は体液過剰であり，進行とともにシャント流量が増加し，さらにシャントが発達して発症するという説がある [16]．われわれの症例でも 4 例中 2 例が維持透析患者であったが，脾静脈と腎静脈は平行して走行しており元々小さな吻合がある可能性があり，腎不全の進行に伴って腎血流が低下し低下した腎静脈圧と脾静脈圧に差が生じシャントが発展するのではないかと考えられる．透析によってその圧格差が広がれば意識障害などの誘発因子になる可能性がある．透析により急激な循環血液量の減少をもたらすことで中心静脈圧が低下し門脈から血液が流入しやすくなることも以前指摘した [4]．

一方，Fischer らによって提唱された分岐鎖アミノ酸（branched chain amino acids:BCAA）の減少と芳香族アミノ酸（aromatic amino acids:AAA）の増加が要因のひとつとされている偽性神経伝達物質説による検討から [19]，門脈大循環短絡路による意識障害を発症している場合，アンモニア値だけでなく，Fischer 比の測定も有用であると考えられる．ICG 値は肝機能を反映する検査であり，肝有効血流量が減少した場合や肝細胞摂取能が低下した場合に，ICG の血中消失速度は遅延する．今回は症例 4 のみしか測定してはいないが，塞栓術前後に測定することにより，肝有効血液量の変化を追うことが可能であると考えられ，ICG 値の測定も治療前後の効果判定には有用であると考えられる．しかし，長期間放置された症例の場合は，肝血流量の低下による肝機能の低下が関与し，正常化しなかった例も報告されており [20]，Fischer 比や ICG 値のみで判定することはできない．

短絡路を閉塞する方法として，外科的に手術する以外にコイルやバルーンを用いてシャントを塞栓する interventional radiology (IVR) 法が行われるようになってきている. 特に B-RTO は他の治療法に比し，低侵襲であり, 根治治療の第一選択となってきている [21]. B-RTO 施行症例の長期管理の問題点としては，血栓化したシャントの再開通，新たな短絡路の形成が挙げられ，治療後も引き続き注意深い経過観察を要する．

症例数としては少なく，まれな疾患ではあるが，短絡路の閉鎖で症状が改善する疾患であり，意識状態や認知障害の程度が変化する患者では鑑別疾患のひとつに挙げるべきだと思われる．高アンモニア血症を証明することは，スクリーニングにおいては特に重要であり，腹部画像精査につなげることができうる．様々な科から紹介される意識障害や浮動性の認知機能障害を有する症例のごく一部にこうした症

例が含まれ神経内科にコンサルトされうるので注意が必要である.

Acknowledgments
We would like to express our sincere appreciation to the late professor emeritus Dr. Hiroko Yamamoto for her support.

Conflict of interest
None.

Funding:
No funding was provided.

文献

1. Watanabe A. Portal-systemic encephalopathy in non-cirrhotic patients: classification of clinical types, diagnosis and treatment. J Gastroenterol Hepatol 2000; 15: 969-79.

2. Shibata A, Hayashi S, Noguchi M. Successful balloon-occluded retrograde transvenous obliteration (B-RTO) in a case of symptomatic epilepsy with hepatic encephalopathy due to non-cirrhotic porto-systemic shunt. Rinsho Shinkeigaku 2005; 45: 341-5 (in Japanese).

3. Miyata K, Tamai H, Uno A, Nakao R, Muroki T, Nasu T, Kawashima A, Nakao T, Kondo M, Ichinose M. Congenital portal systemic encephalopathy misdiagnosed as senile dementia. Intern Medcine 48: 321-4.

4. Kondo N, Ito Y, Yamashita H, Azuma F, Nokura K, Yasuda T, Sobue G. Hemodialysis-related Portal-Systemic Encephalopathy. Internal Medicine 015; 54: 1113-7.

5. The Japan Society for Portal Hypertension. The general rules for study of portal hypertension. 2nd ed. Tokyo: Kanehara shuppan; 2004 (in Japanese).

6. Ferenci P, Lockwood A, Mullen K, Tarter R, Weissenborn K, Blei AT. Hepatic encephalopathy--definition, nomenclature, diagnosis, and quantification: final report of the working party at the 11th World Congresses of Gastroenterology, Vienna, 1998. Hepatology 2002; 35: 716-21.

7. Edwards EA. Functional anatomy of the portal-systemic communications. AMA Arch Intern Med 1951; 88: 137-54.

8. Reichardt W, Butzow GH, Erbe W. Anomalous venous connections involving the portal system. Cardiovasc Radiol 1979; 2: 41-6.

9. Moncure AC, Waltman AC, Vandersalm TJ, Linton RR, Levine FH, Abbott WM. Gastrointestinal hemorrhage from adhesion-related mesenteric varices. Ann Surg 1976; 183: 24-9.

10. Akahoshi T, Nishizaki T, Wakasugi K, Mastuzaka T, Kume K, Yamamoto I, Sugimachi K. Portal-systemic encephalopathy due to a congenital extrahepatic portosystemic shunt: three cases and literature review. Hepatogastroenterology 2000; 47: 1113-6.

11. Walker CO, Schenker S. Pathogenesis of hepatic encephalopathy--with special reference to the role of ammonia. Am J Clin Nutr 1970; 23: 619-32.

12. Ishizaki Y, Kawasaki S. Portosystemic shunts. Japanese Journal of Clinical Medicine 2010; Bessatsu kan tandokei shokogun: 58-63 (in Japanese).

13. Shimizu Y, Kusano M. Portosystemic shunts. Acta Hepatologica Japonica 2002; 43: 479-81 (in Japanese).

14. Shimono J, Tsuji H, Azuma K, Hashiguchi M, Fujishima M. Recurring encephalopathy abolished by gastrorenal shunt ligation in a diabetic hemodialysis patient. Am J Gastroenterol 1998; 93: 270-2.

15. Teramura T, Ishiyama T, Miura Y. Hijomyaku hidarijinjomyaku shanto niyoru hikankohensei himommyakuatsukoshinsei portal-systemic encephalopathy o hasshoshita ketsueki toseki kanja no 1rei. The Japanese Journal of Clinical Dialysis 1999; 15: 757-61 (in Japanese).

16. Yoshimitsu T, Hirakata H. Hikankohensei himommyakuatsukoshinsei chronic portal-systemic shunt encephalopathy(CPSE) o hasshoshita mansei ketsueki toseki kanja no 1rei. The Japanese Journal of Clinical Dialysis 2001; 17: 1387-92 (in Japanese).

17. Ubara Y, Hoshino J, Tagami T, Sawa N, Katori H, Takemoto F, Matsuda M, Hara S, Takaichi K. Hemodialysis-related portal-systemic encephalopathy. Am J Kidney Dis 2004; 44: e38-42.

18. Kondo M, Yamamoto H, Ikeda M, Horiguchi M, Utsunomiya Y, Hosoya T. Hyperammoniemia due to portal-systemic shunt developed after initiation of hemodialysis in a chronic renal failure patient. Nihon Naika Gakkai Zasshi 2004; 93: 1637-8 (in Japanese).

19. Fischer JE, Rosen HM, Ebeid AM, James JH, Keane JM, Soeters PB. The effect of normalization of plasma amino acids on hepatic encephalopathy in man. Surgery 1976; 80: 77-91.

20. Hashimoto K, Takahashi H, Watanabe F, Hasumura S, Aizawa Y, Toda G, Yamada T, Mogami T. A case of gastro-renal-shunt induced non-cirrhotic recurrent hepatic encephalopathy effectually treated by balloon-occluded retrograde transvenous obliteration. Nihon Shokakibyo Gakkai Zasshi 2001; 98: 42-7.

21. Ishiwatari H, Hisai H, Kanisawa Y, Akiyama T, Miyanishi K, Takahashi M, Kato J, Niitsu Y. Two cases of non-cirrhotic porto-systemic shunt successfully treated by balloon-occuluded retrograde transvenous obliteration including evaluation of serial liver biopsy findings. Kanzo 2002; 43: 498-506 (in Japanese)

図の説明

図 1 : 症例 4 のアンモニア値の変化を示す．同病日で大きな日内変動を示しているのは透析前後であり，透析後にはアンモニア値が低下している．図内矢印は embolization を示す．塞栓術後は著明な上昇を認めなくなった．

図 2 : 症例 4 の腹部単純 CT と 3DCT angiography．脾静脈から左腎静脈に異常血管を認めた．

図 3 : 症例 4 のバルーン閉塞下逆行性経静脈的塞栓術（balloon-occluded retrograde transvenous obliteration;B-RTO）前後の血管造影．塞栓術により治療前に認めていた異常血管は描出されなくなった．文献 4 の許可を得て再掲．
